# Etiologies of outpatient medically attended acute respiratory infections among young Ecuadorian children prior to the start of the 2020 SARS‐CoV‐2 pandemic

**DOI:** 10.1111/irv.13056

**Published:** 2022-09-29

**Authors:** Manika Suryadevara, Freddy Pizarro Fajardo, Cinthya Cueva Aponte, Jorge Luis Carrillo Aponte, Esteban Ortiz Prado, Ivan Hidalgo, Cynthia A. Bonville, Irene Torres, Joseph B. Domachowske

**Affiliations:** ^1^ Department of Pediatrics SUNY Upstate Medical University Syracuse New York USA; ^2^ Research Center at Hospital Teófilo Dávila SUNY Upstate Medical University Machala Ecuador; ^3^ One Health Global Research Group Universidad de las Americas Quito Ecuador; ^4^ Centro Medico Hidalgo Jaramillo Quito Ecuador; ^5^ Fundación Octaedro Quito Ecuador

**Keywords:** Ecuador, enterovirus D68, influenza, respiratory infection, RSV

## Abstract

**Background:**

Implementation of respiratory virus prevention measures requires detailed understanding of regional epidemiology; however, data from many tropical countries are sparse. We describe etiologies of ambulatory pediatric acute respiratory tract infections (ARTI) in Ecuador immediately preceding the onset of the SARS‐CoV‐2 pandemic.

**Methods:**

Children < 5 years presenting to a designated study site with an ARTI were eligible. Informed consent was obtained. Demographic and clinical data were recorded. A nasopharyngeal swab was collected, processed, and analyzed using multiplex polymerase chain reaction (PCR) for common respiratory pathogens. Rhinovirus/enterovirus positive samples were further characterized by genomic sequencing.

**Results:**

A total of 820 subjects were enrolled in the study between July 2018 and March 2020. A total of 655 (80%) samples identified at least one pathogen. Rhinoviruses (44%) were most common, followed by enteroviruses (17%), parainfluenza viruses (17%), respiratory syncytial virus (RSV) (15%), and influenza viruses (13%). Enterovirus D68 was the most common enterovirus detected and was among the leading causes of bronchiolitis. Seasonal RSV and influenza virus activity were different along the coast compared with the highlands.

**Conclusions:**

Ongoing regional surveillance studies are necessary to optimize available and emerging pathogen‐specific preventative measures.

## 1 BACKGROUND

Pediatric acute respiratory tract infections (ARTI) cause a substantial disease burden worldwide with children living in countries of lower sociodemographic indices experiencing the highest morbidity and mortality.^1^, ^2^, ^3^, ^4^ Disease prevention strategies are used more widely in developed countries despite higher rates of childhood morbidity and mortality in underdeveloped nations.^5^, ^6^ For example, influenza vaccines are readily accessible in developed countries, yet challenges with availability and distribution often limit widespread use in lower income countries.^7^, ^8^ Ideally, influenza vaccine recommendations should be based on local influenza surveillance data, but such data are often limited.^9^ Similarly, passive immunization against respiratory syncytial virus (RSV), with palivizumab, is safe and effective in preventing severe RSV disease among high‐risk infants, yet its cost precludes its use throughout most of the developing world where RSV‐associated morbidity and mortality are highest.^10^ Cost aside, the lack of reliable longitudinal seasonal data across much of the tropics further challenges efforts to develop robust national public health programs aimed at preventing RSV. Optimal planning and implementation from season to season requires detailed understanding of regional RSV epidemiology.

Ecuador's ARTI disease burden and economic impact were recently estimated to include 14.8 million cases and 17,757 deaths over 5 years,^11^ with associated annual costs during childhood exceeding $50 million.^12^ Where resources permit, the emergence and availability of multiplex polymerase chain reaction (PCR)‐based diagnostics have substantially improved the overall quality of pathogen‐specific surveillance data.^13^, ^14^, ^15^, ^16^ Prior reports on the specific causes of ARTI in Ecuadorian children focused on hospitalized children living in the highlands with a paucity, or complete absence of virus seasonality along the coast.^14^, ^15^, ^17^ Here, we report interim results from our 5‐year surveillance study of the etiologies of ARTI in children enrolled during outpatient sick visits at clinics located in Quito (highlands) and Machala (coastal) during the period immediately preceding the onset of the SARS‐CoV‐2 pandemic.

## 2 METHODS

This 5‐year cross‐sectional surveillance study of the etiologies of medically attended outpatient ARTI among Ecuadorian children < 5 years began in July 2018. Subjects are recruited from outpatient facilities in Quito and in Machala. Enrollment was halted temporarily in March 2020 due to restrictions imposed by the SARS‐CoV‐2 pandemic. Here, we report interim results from July 2018 through March 2020. The project is approved by the SUNY Upstate Medical University Institutional Review Board, by the Bioethics Committee at the University of San Francisco in Quito, and by the Ministry of Health of Ecuador.

### 2.1 Study population

Eligibility for inclusion and recruitment logistics were previously described.^18^ Briefly, children < 5 years of age presenting to a study‐designated ambulatory clinic with a suspected ARTI are eligible. For study purposes, an ARTI is defined as the presence or parental report of two or more of the following for fewer than 8 days: temperature ≥ 38°C, nasal congestion/discharge, cough, tachypnea, wheezing, rales, hypoxia, or apnea. Children in foster care, those with parents unable/unwilling to provide consent, and those hospitalized or treated with antibiotics within the last 30 days are excluded. After obtaining informed consent, demographic and clinical data are recorded, including the assigned primary medical diagnosis for the visit. For study purposes, upper respiratory tract infections (URI) include nasopharyngitis, laryngotracheitis, and influenza‐like illness; lower respiratory tract infections (LRTI) include bronchitis, bronchiolitis, and pneumonia.

A nasopharyngeal swab was collected into 3 ml of universal transport media, processed, and tested using multiplex PCR‐based BioFire FilmArray Respiratory Panel v1.7 (BioFire Diagnostics, LLC, Salt Lake City, UT, USA), allowing for detection of adenoviruses, coronaviruses (HKU1, NL63, 229E, OC43), human metapneumovirus (hMPV), human rhinoviruses/enteroviruses, influenza viruses (IVs) (A‐H1N1, A‐H3N2, B), parainfluenza viruses (PIVs) (1–4), RSV, *Bordetella pertussis*, *Chlamydophila pneumoniae*, and *Mycoplasma pneumoniae*.

The system includes six sets of primer pairs designed to amplify segments of rhinoviruses and enteroviruses; however, RNA sequence similarities between the two groups limit the ability to reliably differentiate one from the other. If any of the six primer pairs amplify their target, the reported result is “detection of human rhinovirus/enterovirus” (BioFire® Respiratory Panel Instructions for Use; https://www.biofiredx.com/support/documents/#toggle-id-3). Next, we further characterized rhinovirus/enterovirus group positive samples using the VIASURE Rhinovirus+Enterovirus Real‐Time PCR Detection (CerTest Biotec, Zaragoza, Spain), according to the manufacturer's instructions. This assay detects and differentiates between rhinovirus‐ and enterovirus‐specific RNA. Briefly, extracted RNA is subjected to reverse transcription and then amplified by targeting a highly conserved sequences of the 5'UTR of rhinoviruses/enteroviruses and using virus‐specific primer pairs. Fluorescence‐labeled probes included in the amplification mix allow the user to distinguish whether a detected signal represents sequence specific to rhinovirus or enterovirus. Positive, negative, and internal controls were included with each run. All enterovirus positive samples were then further characterized by genomic sequencing. BLAST algorithm software (4 Peaks for Mac v. 1.8, Softonic, Barcelona, Spain) was used to compare sequencing results with known nucleotide sequences available in the National Center for Biotechnology Information (NCBI) database.

Descriptive statistics were used. Categorical data were compared using the Fisher exact or chi‐square test, as appropriate. Significance was set a priori to p‐values < 0.05.

## 3 RESULTS

**FIGURE 1:**
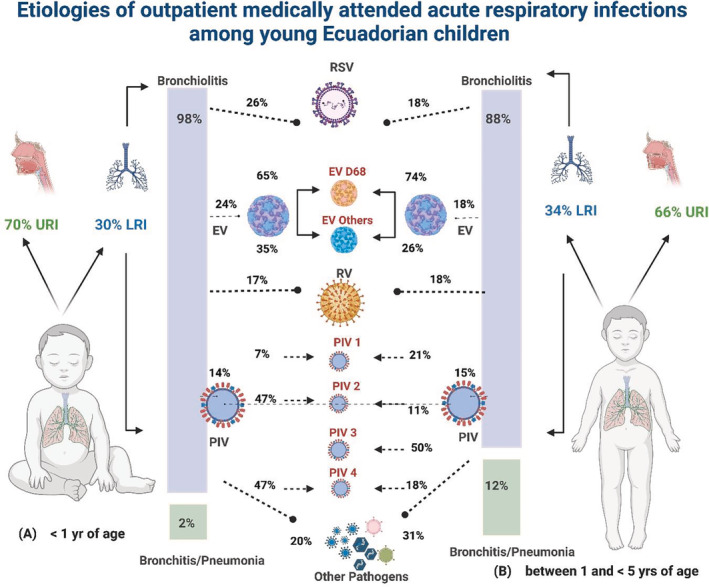
Etiologies of outpatient medically attended acute respiratory infections among young Ecuadorian children. EV, enterovirus; LRI, lower respiratory tract infections; PIV, parainfluenza virus; RSV, respiratory syncytial virus; URI, upper respiratory tract infections

A total of 820 subjects were enrolled through March 2020: 491 (60%) in Machala and 329 (40%) in Quito. The median age was 18 months, with 301 (37%) subjects <1 year of age and 519 (63%) between 1 and 5 years. Subjects enrolled in Quito were older (30 months vs. 13 months; p < 0.05) and had higher mean rates of vaccination against influenza (65% vs. 56%, p < 0.05) compared with those enrolled in Machala. Of the 802 children (316 in Quito and 486 in Machala) with clear documentation, 543 (68%) and 261 (33%) were diagnosed with URI and LRTI, respectively. The most frequent URI diagnosis (68%) was nasopharyngitis whereas the most frequent LRTI diagnosis was bronchiolitis (92%). Subjects enrolled in Quito (169/316 [53%]) were more likely to be diagnosed with LRTI compared with those enrolled in Machala (92/486 [19%]) (p < 0.05). Among all subjects with LRTI, a higher proportion were diagnosed with bronchiolitis in Quito (161/169, 95%) than in Machala (77/92, 84%) (p < 0.05). When stratified by age, there was no significant difference in proportion of LRTI diagnosed in children < 1 year compared with those ≥1 year (89/295 [30%] vs. 172/507 [34%], p > 0.05). Subjects < 1 year of age with LRTI were more likely to be diagnosed with bronchiolitis (87/89 [98%]) compared with those between 1 and 5 years (151/172 [88%], p = 0.007) (Figure 1).

Results from the respiratory pathogen multiplex PCR and subsequent analyses, when performed, are shown in Table 1. The most frequently detected pathogen group was rhinovirus (285, 44%) followed by enterovirus (111, 17%), PIVs (113, 17%), RSV (99, 15%), IVs (83, 13%), and hMPV (63, 10%).

**TABLE 1:** Respiratory pathogens detected from study participants by enrollment site

	Total n (%)	Machala n (%)	Quito n (%)	p‐value^a^
Samples collected	820	491	329	
One or more pathogens detected	655 (80)	374 (76)	281 (85)	0.001
Rhinoviruses	285 (44)	184 (49)	101 (36)	<0.001
Enteroviruses	111 (17)	29 (8)	82 (29)	<0.001
Coxsackie A6	12 (11)	3 (10)	9 (11)	0.9
Coxsackie A9	9 (8)	0	9 (11)	
Coxsackie B4	8 (7)	0	8 (10)	
Coxsackie B5	12 (11)	0	12 (15)	
Coxsackie B6	2 (2)	2 (7)	0	0.1
Echovirus 3	3 (3)	0	3 (4)	
Echovirus 11	4 (4)	3 (10)	1 (1)	0.02
Echovirus 18	6 (5)	0	6 (7)	
Enterovirus C105	7 (6)	7 (24)	0	
Enterovirus D68	48 (43)	14 (48)	34 (41)	0.52
Parainfluenza viruses	113 (17)	57 (15)	56 (20)	0.12
Parainfluenza virus type 1	22 (19)	7 (12)	15 (27)	0.52
Parainfluenza virus type 2	9 (8)	4 (7)	5 (9)	0.71
Parainfluenza virus type 3	53 (47)	27 (47)	26 (46)	0.92
Parainfluenza virus type 4	29 (26)	19 (33)	10 (18)	0.06
Respiratory syncytial virus	99 (15)	40 (11)	59 (21)	<0.001
Influenza viruses	83 (13)	38 (10)	45 (16)	0.03
Influenza A H1N1	10 (12)	6 (16)	4 (9)	0.34
Influenza A H3N2	34 (41)	11 (29)	23 (51)	0.04
Influenza B	37 (45)	19 (50)	18 (40)	0.36
Influenza virus—not typeable	2 (2)	2 (5)	0	
Human metapneumovirus	63 (10)	40 (11)	23 (8)	0.28
Adenovirus	55 (8)	20 (5)	35 (12)	0.001
Coronaviruses	44^b^ (7)	27^b^ (7)	17 (6)	0.55
Coronavirus OC43	15 (34)	6 (22)	9 (53)	0.04
Coronavirus HKU1	6 (14)	3 (11)	3 (18)	0.54
Coronavirus 229E	11 (25)	8 (30)	3 (18)	0.37
Coronavirus NL63	14 (32)	12 (44)	2 (12)	0.02
Atypical bacteria	15 (2)	11 (3)	4 (1)	0.199
*Mycoplasma pneumoniae*	9 (60)	5 (45)	4 (100)	
*Chlamydia pneumoniae*	6 (40)	6 (55)	0	
*Bordetella pertussis*	0	0	0	
Single pathogen detected	481 (73)	307 (82)	174 (62)	<0.001
Two pathogens detected	153 (23)	66 (18)	87 (31)	<0.001
Three pathogens detected	17 (3)	1 (<1)	16 (6)	<0.001
Four pathogens detected	4 (<1)	0	4 (1)	

^a^ p‐value when comparing the frequency of detection between Machala and Quito.

^b^ Total number does not equal the sum of the virus types as two subjects had co‐detection of two coronavirus types.

**FIGURE 2:**
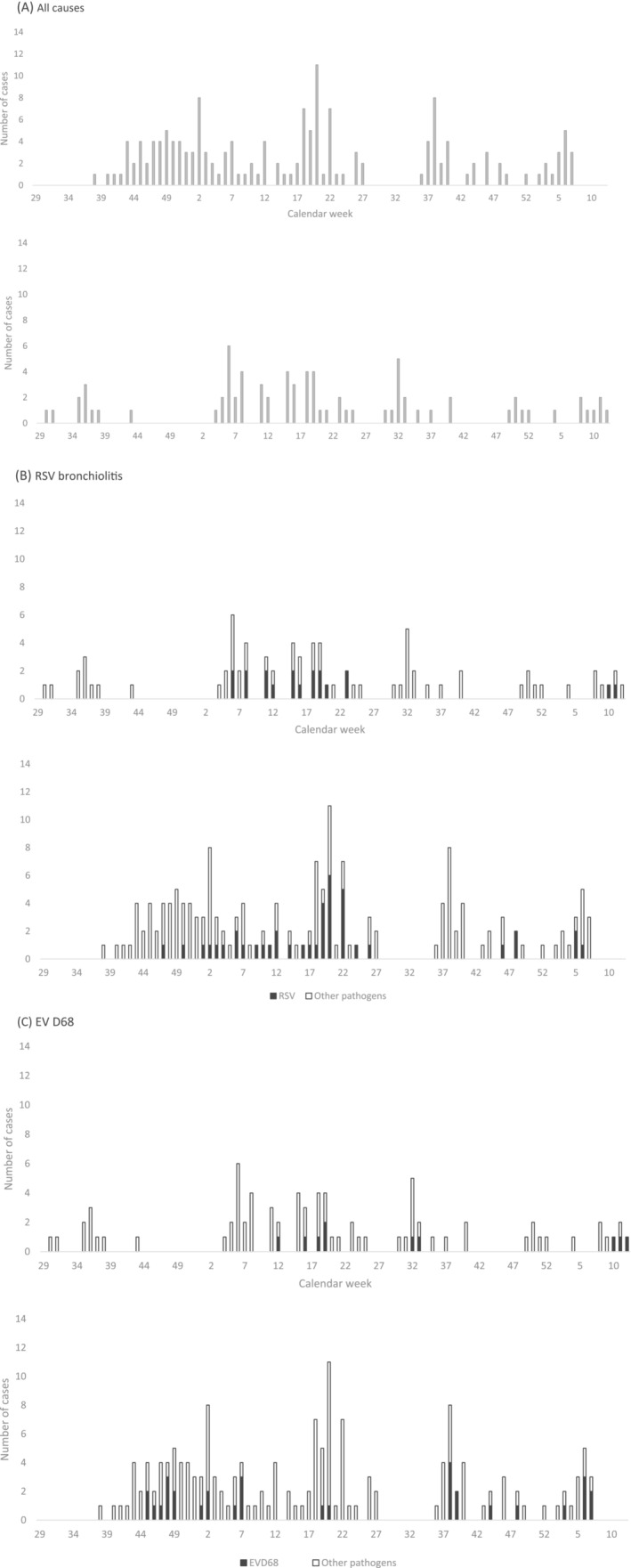
Bronchiolitis cases by enrollment location (top panel, Machala; bottom panel, Quito) and by calendar week from (A) all causes, (B) respiratory syncytial virus (RSV), and (C) enterovirus (EV) D68

Clinical characteristics of subjects by pathogen group detected are summarized in Table 2. Almost half (156/322, 48%) of the respiratory samples collected from subjects diagnosed with nasopharyngitis were positive for the detection of rhinovirus. Clinically, RSV infection was more likely to be associated with wheezing and a clinical diagnosis of LRTI than any other respiratory virus (p < 0.05). Subjects diagnosed with bronchiolitis were identified year‐round (Figure 2). RSV was the most common pathogen identified among the 238 subjects diagnosed with bronchiolitis (61, 26%) followed by enteroviruses (60, 25%), rhinoviruses (51, 21%), and PIVs (43, 18%) (Figure 1), whether or not the subjects were stratified by age. Enteroviruses were more commonly detected among children with bronchiolitis in Quito (48/161, 30%) than in Machala (10/77, 13%) (p < 0.05) (Table 3). Specifically, enterovirus D68 (EV‐D68) was identified in 40/58 (69%) enterovirus positive samples collected from subjects diagnosed with bronchiolitis.

**TABLE 2:** Clinical characteristics of subjects by pathogen group detected

	AdV n (%)	hMPV n (%)	PIV n (%)	RV n (%)	EV n (%)	RSV n (%)	IV n (%)	CoV n (%)	Atypical bacteria n (%)
Number of patients	55	63	113	285	110	99	83	44	15
Median age (months)	25	24	18	17	24	18	27	17	14
Male, n (%)	34 (62)	33 (52)	65 (58)	143 (50)	60 (54)	59 (60)	41 (49)	23 (52)	9 (60)
Mean symptom duration (days)	2.5	3	2.8	2.9	2.4	2.8	2.7	2.6	3.9
**Symptoms**									
Fever	49 (89)	56 (89)	91 (81)	215 (75)	99 (89)	83 (84)	77 (93)	33 (75)	15 (100)
Nasal congestion	55 (100)	63 (100)	106 (93)	278 (98)	111 (100)	99 (100)	81 (98)	42 (95)	11 (73)
Cough	48 (87)	57 (90)	98 (87)	218 (76)	100 (90)	97 (98)	70 (84)	35 (80)	8 (53)
Wheeze	32 (58)	26 (41)	65 (58)	101 (35)	78 (70)	73 (74)	44 (53)	16 (36)	0
**Clinical diagnosis**									
**Upper respiratory infection**	31 (56)	42 (67)	63 (56)	217 (76)	45 (41)	31 (31)	58 (59)	33 (75)	4 (27)
Laryngotracheitis	9 (16)	9 (14)	16 (14)	33 (12)	18 (16)	12 (12)	15 (18)	8 (18)	1 (7)
Nasopharyngitis	21 (38)	31 (49)	14 (12)	156 (55)	23 (21)	14 (14)	40 (48)	20 (45)	3 (20)
Influenza‐like illness	1 (2)	2 (3)	6 (5)	28 (10)	4 (4)	5 (5)	3 (4)	5 (11)	0
**Lower respiratory infection**	25 (45)	21 (33)	46 (41)	61 (21)	65 (59)	67 (68)	24 (29)	12 (27)	10 (67)
Bronchiolitis	22 (40)	19 (30)	43 (38)	52 (18)	60 (54)	61 (62)	22 (27)	10 (23)	8 (53)
Bronchitis	1 (2)	0	2 (2)	4 (1)	0	1 (1)	0	0	0
Pneumonia	2 (4)	2 (3)	1 (1)	5 (2)	5 (5)	5 (5)	2 (2)	2 (5)	2 (13)
Antibiotics prescribed	15 (27)	10 (16)	12 (11)	34 (12)	18 (16)	27 (27)	15 (18)	3 (7)	4 (27)

Abbreviations: AdV, adenovirus; CoV, coronavirus; EV, enterovirus; hMPV, human metapneumovirus; IV, influenza virus; PIV, parainfluenza virus; RSV, respiratory syncytial virus; RV, rhinovirus.

**TABLE 3:** Frequency of pathogen detection associated with bronchiolitis by enrollment site

	Total n (%)	Machala n (%)	Quito n (%)	p‐value
Total	238	77 (32)	161 (68)	
Respiratory syncytial virus	61 (26)	19 (25)	42 (26)	>0.05
Enteroviruses	58 (24)	10 (13)	48 (30)	0.005
Enterovirus D68	40 (69)	10 (100)	30 (63)	0.02
Rhinoviruses	51 (21)	19 (25)	32 (20)	>0.05
Parainfluenza viruses	43(18)	10 (13)	33 (20)	>0.05
Parainfluenza virus type 3	21 (49)	3 (30)	18 (55)	>0.05
Influenza viruses	22 (9)	4 (5)	18 (11)	>0.05
Adenoviruses	22 (9)	8 (10)	14 (9)	>0.05
Human metapneumovirus	19 (8)	5 (6)	14 (9)	>0.05
Seasonal coronaviruses	10 (4)	3 (4)	7 (4)	>0.05
Atypical bacteria	8 (3)	5 (6)	3 (2)	>0.05

In Machala, bronchiolitis cases were diagnosed throughout the study period, with no clear clustering (Figure 2A, top panel). Enterovirus‐associated bronchiolitis occurred between March and May whereas RSV bronchiolitis occurred between February and June. Differences in seasonal patterns of virus activity were observed in Quito where bronchiolitis was diagnosed most consistently between September and July (Figure 2A, bottom panel). Rhinovirus‐ and enterovirus‐associated bronchiolitis was observed throughout the study period. PIV‐associated bronchiolitis was biphasic, clustering between May and July and again between October and January, whereas RSV bronchiolitis occurred from November to May.

### 3.1 Pathogen‐specific results

The most commonly detected pathogen group, accounting for 285/655 (44%) positive samples, were the rhinoviruses (Table 2). The majority of rhinovirus‐positive subjects (217, 76%) were diagnosed with a URI, most commonly nasopharyngitis (156, 55%). Rhinoviruses were more likely to be detected in the samples collected from children enrolled in Machala (184, 49%) than in Quito (101, 36%) (p < 0.05), and cases were detected year‐round, without clear seasonality at either site.

Enteroviruses ranked second, detected in 111 (17%) positive samples (Table 2). Of these, 45 (41%) study participants were diagnosed with a URI, most commonly nasopharyngitis (23, 51%). Sixty‐five (59%) enterovirus positive subjects were diagnosed with an LRTI, 60 (92%) of whom had bronchiolitis. EV‐D68 predominated, accounting for 48% and 41% of enteroviruses detected in samples collected in Machala and Quito, respectively. Subjects who tested positive for EV‐D68 were more likely to have documented wheezing than those who tested positive for other enteroviruses (42/48 [88%] vs. 36/63 [57%], p < 0.05). In Quito, wheezing was observed in 34/34 (100%) of EV‐D68 positive subjects and 35/48 (73%) of those positive for an enterovirus other than EV‐D68 (p < 0.05). In Machala, enterovirus‐associated wheezing was less frequent but remained more common among those testing positive for EV‐D68 (8/14 [57%]) than for all other enteroviruses combined (1/36 [3%]; p < 0.05).

**FIGURE 3:**
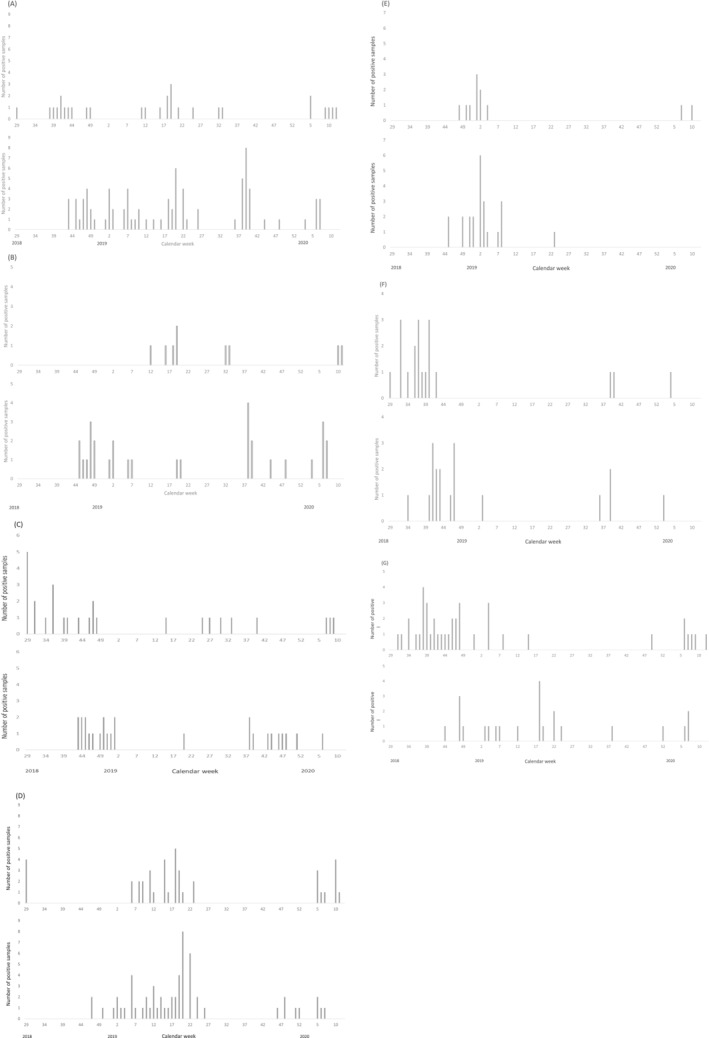
Detection of select respiratory pathogens by week at enrollment site. (A) Enteroviruses, (B) enterovirus D68, (C) parainfluenza virus type 3, (D) respiratory syncytial virus, (E) influenza A H3N2 virus, (F) influenza B virus, and (G) human metapneumovirus. For each set, the top and bottom panels represent data from Machala and Quito, respectively.

PIVs were detected in 113 (17%) positive samples (Table 2). Almost half of the PIVs (53, 47%) detected were identified as PIV type 3 (Table 1). Sixty‐three (56%) and 46 (41%) of all PIV‐positive subjects were diagnosed with URI and LRTI, respectively. The most common URI manifestations were laryngotracheitis (16, 14%) and nasopharyngitis (14, 12%), whereas the most common LRTI manifestation was bronchiolitis (43, 38%). PIV type 3 accounted for 21/238 (8.8%) subjects diagnosed with bronchiolitis and for 21/43 (49%) of all PIV‐associated cases of bronchiolitis. As a group, PIVs were detected year‐round. However, evaluation of individual PIV types by site showed a clear seasonal pattern only for PIV type 3 activity in Quito, where detection peaked between the months of October and December (Figure 3). In contrast, a clear seasonal peak of PIV type 3 activity was not observed in Machala.

RSV was detected in 99 (15%) samples (Table 2). Thirty‐one (31%) and 67 (68%) RSV positive subjects were diagnosed with URI and LRTI, respectively. The most common URI manifestations were nasopharyngitis (14, 45%) and laryngotracheitis (12, 39%); the most common LRTI manifestation was bronchiolitis (61, 91%). Overall, RSV was the most common pathogen associated with a diagnosis of bronchiolitis, accounting for 61/238 (26%) of all bronchiolitis cases. In Machala, detection of RSV was tightly clustered between February and May 2019, reappearing again in February 2020. RSV detection was ongoing into March 2020 when study activities were halted (Figure 3). In contrast, RSV detection was first noted in Quito during mid‐November 2018, with more steady activity continuing during January and February 2019. The number of weekly positive samples tapered until the end of June. No samples tested positive for RSV between July and mid‐November 2019 when low levels of activity resumed and continued until enrollment was halted in March 2020.

IVs were detected in 83 (13%) nasopharyngeal samples (Table 2). Of these, 58 (70%) and 24 (29%) subjects were diagnosed with URI and LRTI, respectively. The most common URI manifestation was nasopharyngitis (40, 69%) whereas the most common LRTI manifestation was bronchiolitis (22, 92%). Despite a 9% higher rate of influenza vaccination, subjects enrolled at the Quito site were more likely to test positive for influenza when compared with those enrolled in Machala (16% vs. 10%, p < 0.05). During the study period, influenza B (37, 45%) and influenza A H3N2 (34, 41%) were the most common subtypes detected (Table 1). In Quito, approximately half of IVs detected were identified as influenza A H3N2 (23, 51%), whereas this virus subtype accounted for less than one third of IVs detected in samples collected in Machala (11, 29%) (p < 0.05).

Clear and overlapping seasonal patterns for IV circulation were observed between Machala and Quito. In Machala, influenza cases were detected between July 2018 and January 2019, then sporadically between September 2019 and the remainder of the study period. In Quito, influenza cases were noted between August 2018 and February 2019, then sporadically between June 2019 and the remainder of the study period. Influenza A H3N2 predominated between November and February, whereas positive tests for influenza B viruses were observed between July and November (Figure 3).

Adenoviruses, hMPV, and seasonal coronaviruses each accounted for 10% or less of the pathogens detected from nasopharyngeal samples (Table 1), yet collectively, they accounted for 59 of the 238 (25%) cases of bronchiolitis. The clinical and demographic distribution of children testing positive for detection of seasonal coronaviruses has been previously published.^18^

## 4 DISCUSSION

This is among the first reports to prospectively describe respiratory pathogen epidemiology among young Ecuadorian children seeking ambulatory medical care for an ARTI. The most frequently identified respiratory pathogens included rhinoviruses (44%), enteroviruses (17%), PIVs (17%), RSV (15%), and IVs (13%). Eighty percent of samples from our study tested positive for one or more pathogens. Others have reported a positive virus detection rate < 30% using PCR to detect IVs, along with the less sensitive direct immunofluorescence assay for other viruses,^15^ demonstrating the importance of using more sensitive diagnostics when high‐quality surveillance results are desired.^19^, ^20^

Almost one third of children enrolled in our study were diagnosed with bronchiolitis, with enteroviruses, RSV, and PIV type 3 identified as the leading causes. EV‐D68 accounted for 70% of enterovirus‐associated bronchiolitis during the study period. This respiratory pathogen is a known cause of severe pediatric LRTI with wheezing but, until now, was not previously described from Ecuador.^21^

Using molecular diagnostics, Jonnalagadda *et al* reported detection of at least one respiratory pathogen in 73% of 406 children hospitalized with severe pneumonia in Quito. RSV, hMPV, adenoviruses, and IVs were most commonly detected in that cohort,^14^ although the diagnostic assays used did not include rhinoviruses, enteroviruses, or coronaviruses. It is important to note that despite differences in illness severity from subjects included in that study and in this report, both sets of data clearly demonstrate the contribution of RSV and IVs on ARTI burden.

Azziz‐Baumgartner *et al* previously reported that >20% of Ecuadorian children aged 12–23 months with a medically attended ARTI tested positive for RSV using immunofluorescence, with infection rates higher than those seen in high‐income countries.^13^ Despite the reported high rates, their findings likely reflect an underestimate of overall disease burden due to the lower sensitivity of immunofluorescence assays compared with PCR‐based diagnostics. Consistent with our work, a majority of young children infected with RSV were diagnosed with LRTI, whereas subjects infected with other respiratory viruses were more likely to be diagnosed with URI. Similarly, subjects who test positive for RSV were more likely to wheeze than those testing positive for other viruses.

Promising emerging strategies for RSV prevention, including extended half‐life monoclonal antibodies, could theoretically be administered to all infants at the start of their first RSV season. Estimates suggest a period of protection lasting 5 months or longer.^22^ Optimal implementation will require detailed knowledge of known and expected RSV seasonality, along with ongoing surveillance to follow patterns of outbreaks prospectively.

During our efforts, we identified 10 different enteroviruses circulating as causes of ARTI, with EV‐D68 predominating. Each has previously been reported as a cause of ARTI, at times with other disease manifestations.^23^, ^24^, ^25^, ^26^ Among the enteroviruses detected, we identified small clusters of infection caused by enterovirus C105 at both study sites. This somewhat rare enterovirus genotype emerged during the last decade and is now presumed to have global distribution, causing predominantly respiratory and/or gastrointestinal symptoms, with at least one case reported in association with acute flaccid paralysis.^27^, ^28^, ^29^

There are currently little publicly available and/or published data regarding IV epidemiology from the existing surveillance systems in Ecuador. Data published a decade ago are consistent with our findings here that show year‐round IV activity.^17^ Understanding recent and current IV epidemiology is necessary to optimize disease prevention strategies. Although influenza vaccine recommendations across equatorial regions should be determined by regional epidemiology and active surveillance, such data are often unavailable, leaving public health decisions on the timing and formulation of annual vaccine programs to be made based solely on geographic location. Results from this study, which continues through the calendar year 2023, and future efforts that focus on factors that help to explain differing circulation patterns of respiratory virus activity across different areas of Ecuador and other equatorial communities are increasingly important as new and emerging prevention measures become available.

## CONFLICTS OF INTEREST

The authors have no conflicts of interest to declare.

## AUTHOR CONTRIBUTIONS

**Manika Suryadevara**: Conceptualization; data curation; formal analysis; investigation; methodology; visualization; writing—original draft preparation; writing—review and editing. **Freddy Pizarro Fajardo**: Data curation; investigation; methodology; writing—review and editing. **Cinthya Cueva Aponte**: Data curation; investigation; methodology; writing—review and editing. **Jorge Luis Carrillo Aponte**: Data curation; investigation; methodology; writing—review and editing. **Esteban Ortiz Prado**: Data curation; investigation; methodology; writing—review and editing. **Ivan Hidalgo**: Data curation; investigation; methodology; writing—review and editing. **Cynthia A. Bonville**: Data curation; investigation; methodology; visualization; writing—review and editing. **Irene Torres**: Data curation; investigation; methodology; writing—review and editing. **Joseph B. Domachowske**: Conceptualization; data curation; formal analysis; investigation; methodology; project administration; resources; supervision; validation; visualization; writing—original draft preparation; writing—review and editing.

### PEER REVIEW

The peer review history for this article is available at https://publons.com/publon/10.1111/irv.13056.

## Data Availability

The data that support the findings of this study are available from the corresponding author upon reasonable request.
